# Negative Pressure Wound Therapy Instillation for Management of Intrathoracic Chronic Infection

**DOI:** 10.1097/GOX.0000000000002323

**Published:** 2019-07-29

**Authors:** Haitham H. Khalil, Ehab Bishay, Marco N. Malahias, Sherif Youssef, Saif Rhobaye, Tarek Ashour, Platon Trigkatzis, Maninder Kalkat

**Affiliations:** From the * Plastic and Reconstructive Surgery Division, Good Hope Hospital, University Hospitals Birmingham Trust, Rectory Road, Sutton Coldfield, Birmingham, B75 7RR, West Midlands, United Kingdom; † Department of Thoracic Surgery, Heartlands Hospital, University Hospitals Birmingham, Bordesley Green East, Birmingham, B95SS, West Midlands, United Kingdom.

Management of chronic intrathoracic infections (empyema) represent a challenging problem in modern thoracic surgery with overall mortality reaching 26%.^1^ Traditionally, several techniques have been performed to control the infection including chest tube insertion, thoracoscopic, or open debridement; however, results in the majority of cases were disappointing.^1^ It is noteworthy that currently the most widely practised and accepted treatment would be open window thoracostomy via rib resection and open drainage as a preliminary procedure or definitive treatment.^1,2^ Recently, vacuum-assisted closure (VAC) therapy has been introduced for management of intrathoracic infection successfully.^2,3^ Respectively, Sziklavari et al.^4^ introduced instillation to the VAC therapy through a mini access approach using mini-VAC instill and antiseptic solution to control infection confined only to the intrathoracic cavity. The authors have expanded its indications to manage 2 patients with intrathoracic chronic infection associated with complex geometric wounds and tracts communicating with the thoracic and anterior abdominal wall. The V.A.C. Ulta device (Kinetis Concepts, Inc, San Antonio, Tex.) was used for negative pressure wound therapy instillation (NPWTi) following open window thoracostomy, wound debridement, and application of Verflow dressing which would be the first to be reported in this context. Both patients had other alternative options exhausted and they were surgically unfit for major reconstructive procedures. In addition, the poor quality of local and regional tissue due to the previous history of thoracic and abdominal scars has hindered the feasibility of any local reconstructive produces not feasible. The details of the operative technique and outcomes are demonstrated in a video (See Video, [online], which demonstrates the management of seventy 5-year-old patients refereed with acute exacerbation of sepsis from right chronic intrathoracic infection (empyema) communicating with thoracic and anterior abdominal wall using NPWTi). Frequency of the dressing change was principally determined by the contamination load and the wound microbiology culture swab results. Initially, it was performed under general anaesthesia and subsequently on ward as in concordance with other studies.^1^ The advent of adding instillation provides an additional dimension with the ability to deliver a solution to the wound in a programmed manner. Although the primary benefit of VAC is the promotion of wound healing through enhancement of granulation tissue and obliterating any dead space, there is some evidence that it may inhibit bacterial growth and reduce infection.^5^ On the other hand, the principle benefits of saline or antiseptic solution for irrigation of infected wounds has been well established in the literature.^5^ Interestingly, combining instillation with VAC therapy enhances the effectiveness of both of the modalities.^5^ This approach was mainly used as a bridge for reconstructive surgery or delayed primary closure as also shown in previously reported studies.^4,5^ NPWTi has not been recommended in thoracic and abdominal cavities due to potential risk of alteration of body temperature.^5^ However, from our observation this was not experienced possibly because of the chronic nature of the infection and the development of chronic scarring. That being said, NPWTi would open potential doors for successful treatment of complex chronic intrathoracic infection in high-risk patients and would be an adjunct in the armamentarium for management of this challenging cohort of patients.

**Video 1.** This video demonstrates the management of seventy 5-year-old patients refereed with acute exacerbation of sepsis from right chronic intrathoracic infection (empyema) communicating with thoracic and anterior abdominal wall using NPWTi. The Verflow cleanse foam and V.A.C. ulta device was used to achieve a quick and efficient cleansing of the wound post debridement, avoiding recolonization, limiting cross-contamination, and aerosolization. This process was used as a bridge before reconstructive procedure using split thickness graft. In this case, the instillation volume was 30 ml saline infused over 10 minutes followed by negative suction 3 hours. This cycle was repeated regularly over 72 hours which is the duration between every change of dressing. One-year follow-up; the patient showed no evidence of recurrence of the infection or sepsis.

## Acknowledgment

The authors would like to thank Jamie Ryan-Ainslie and the entire Medical Illustration Team at University Hospitals Birmingham (UHB) for their expert input.
